# Care Transitions of Colorectal Cancer Patients from Hospital to Community: Systematic Review and Meta-analysis Protocol

**DOI:** 10.15649/cuidarte.2285

**Published:** 2021-08-20

**Authors:** Julia Estela Willrich-Boell, Letícia Flores-Trindade, Adriane Cristina Bernat-Kolankiewicz, Wilson Cañon-Montañez, Edith Pituskin, Elisiane Lorenzini

**Affiliations:** 1 RN, PhD. Federal University of Santa Catarina, Florianópolis, SC, Brazil. E-mail: juliasuporte@gmail.com Federal University of Santa Catarina Florianópolis Brazil juliasuporte@gmail.com; 2 RN, MN. Regional University of the Northwest of the State of Rio Grande do Sul, Ijuí, Rio Grande do Sul, Brazil. E-mail: leti.ftrindade@yahoo.com.br Regional University of the Northwest of the State of Rio Grande do Sul Brazil leti.ftrindade@yahoo.com.br; 3 RN. PhD. Regional University of the Northwest of the State of Rio Grande do Sul, Ijuí, Rio Grande do Sul, Brazil. E-mail: adriane.bernat@unijui.edu.br Regional University of the Northwest of the State of Rio Grande do Sul Brazil adriane.bernat@unijui.edu.br; 4 RN. PhD. Faculty of Nursing, Universidad de Antioquia, Medellín, Antioquia, Colombia. E-mail: wilson.canon@udea.edu.co Universidad de Antioquia Universidad de Antioquia Medellín Colombia wilson.canon@udea.edu.co; 5 RN, MN, PhD. Faculty of Nursing University of Alberta, Edmonton, Alberta, Canada. E-mail: pituskin@ualberta.ca University of Alberta University of Alberta Canada pituskin@ualberta.ca; 6 RN. PhD. Federal University of Santa Catarina, Florianópolis, Santa Catarina, Brazil. E-mail: elisiane.lorenzini@ufsc.br Universidade Federal de Santa Catarina Federal University of Santa Catarina Florianópolis Brazil elisiane.lorenzini@ufsc.br

**Keywords:** Colorectal neoplasms, Patient transfer, Patient readmission, Systematic review, Meta-analysis., Neoplasias colorrectales, Transferencia de pacientes, Readmisión del paciente, Revisión sistemática, Metaanálisis., Neoplasias colorretais, Transferência de pacientes, Readmissão do paciente, Revisão sistemática, Metanálise.

## Abstract

**Objective:**

To evaluate the effectiveness of care transition strategies from hospital-to-community compared to usual care for patients with colorectal cancer to reduce hospital stay, 30-day readmissions, and emergency room visits up to 30 days.

**Methods:**

Systematic review and meta‐analysis protocol that followed the recommendations of the Preferred Reporting Items for Systematic Reviews and Meta-Analyses Protocols (PRISMA-P). The protocol was registered on PROSPERO (CRD42020162249). We will include studies available in the electronic databases PubMed/Medline, Embase, Cochrane CENTRAL and LILACS with care transition strategies/actions from hospital to community as the primary outcome. Eligible studies will be selected, and data will becombined and synthesized using Review Manager (RevMan 5.4) software. We will combine risk ratios or odds ratios for dichotomous data and mean differences for continuous data using a random effects model.

**Discussion:**

This review will contribute to the practice and development of effective and safe care transition strategies from hospital to community for colorectal cancer patients. There is an expectation that this review will provide much needed evidence that effective care transitions could reduce short term hospital readmission, and may thus provide added value in the care of colorectal cancer patients.

**Conclusion:**

The results of the review will be used to provide clear recommendations for hospital and primary care management to improve care transitions and, as a result, also improve integration in the healthcare system.

## Resumen

**Objetivo:**

Evaluar la efectividad de estrategias de transición de cuidados del hospital a la comunidad en comparación con el cuidado usual en pacientes con cáncer colorrectal para reducir el tiempo de estancia hospitalaria, readmisiones a los 30 días y visitas al departamento de emergencias dentro de los 30 días.

**Métodos:**

Protocolo de revisión sistemática y metaanálisis que siguió las recomendaciones de *Preferred Reporting Items for Systematic Reviews and Meta-Analyses Protocols* (PRISMA-P). El protocolo se registró en PROSPERO (CRD42020162249). Se incluirán estudios disponibles en las bases de datos electrónicas PubMed/Medline, Embase, Cochrane CENTRAL y LILACS con estrategias/acciones de transición del cui- dado del hospital a la comunidad como desenlace principal. Se seleccionarán los estudios elegibles y los datos se combinarán y sintetizarán mediante el software Review Manager (RevMan 5.4). Serán com- binados los riesgos relativos u odds ratios para los datos dicotómicos y las diferencias de medias para los datos continuos mediante un modelo de efectos aleatorios.

**Discusión:**

Esta revisión contribuirá a la práctica y el desarrollo de estrategias de transición de cuidado efectivas y seguras del hospital a la co- munidad para los pacientes con cáncer colorrectal. Se espera que esta revisión proporcione evidencias muy necesarias de que las transiciones de cuidado efectivas podrían reducir la readmisión hospitalaria a corto plazo y, por lo tanto, pueden proporcionar un valor agregado en el cuidado de los pacientes con cáncer colorrectal.

**Conclusión:**

Los resultados de la revisión se utilizarán para proporcionar recomenda- ciones claras para la gestión hospitalaria y de cuidado primario para mejorar las transiciones de cuidado y, como resultado, también mejorar la integración en el sistema de salud.

## Resumo

**Objetivo:**

Avaliar a efetividade de estratégias de transição do cuidado do hospital para a comunidade comparada aos cuidados habituais para pacientes com câncer colorretal para diminuir tempo de per- manência hospitalar, readmissões aos 30 dias e visita ao setor de emergência até 30 dias.

**Métodos:**

Pro- tocolo de revisão sistemática e meta-análise que seguiu as recomendações do *Preferred Reporting Items for Systematic Reviews and Meta-Analyses Protocols* (PRISMA-P). O protocolo foi registrado no PROSPERO (CRD42020162249). Incluiremos estudos disponíveis nas bases de dados eletrônicas PubMed/Medline, Embase, Cochrane CENTRAL e LILACS tendo como desfecho primário as estratégias/ações de transição do cuidado do hospital para a comunidade. Os estudos elegíveis serão selecionados e os dados serão combinados e sintetizados usando o software Review Manager (RevMan 5.4). Serão combinados os riscos relativos ou odds ratios para dados dicotômicos e diferenças de médias para dados contínuos usando um modelo de efeitos aleatórios.

**Discussão:**

Esta revisão contribuirá para a prática e desenvol- vimento de estratégias de transição de cuidados efetivas e seguras do hospital para a comunidade para pacientes com câncer colorretal. Espera-se que esta revisão forneça evidências muito necessárias de que as transições de cuidados efetivas podem reduzir a readmissão hospitalar de curto prazo e podem, assim, fornecer valor agregado no cuidado de pacientes com câncer colorretal.

**Conclusão:**

Os resultados da revisão serão usados para fornecer recomendações claras para a gestão hospitalar e de cuidados primários para melhorar as transições de cuidados e, como resultado, também melhorar a integração no sistema de saúde.

## Introduction

Cancer has taken on large proportions worldwide and its incidence has progressively increased, with high morbidity and mortality(1). Globally, one in five men and one in six women develop cancer during their lifetime, and one in eight men and one in each 11 women die from the disease(1). Worldwide, an estimated 19.3 million new cancer cases and almost 10.0 million cancer deaths occurred in 2020(2). In the Americas in 2020, cancer was responsible for 20.9% of all cases worldwide and for 14.2% of mortality(2). Still, regarding the types of cancers, colorectal is in third place in terms of incidence, and second in terms of mortality(2). More than 1.9 million new cases of colorectal cancer (CRC) and 935,000 deaths occurred in 2020, representing about one in 10 cancer cases and deaths(2).

In developing countries, such as Brazil, limitations on access to healthcare, such as low coverage of screening programs and delays in carrying out diagnostic and therapeutic procedures(3),(4) contribute to the increase of the incidence and mortality from this disease(5). In addition, geographical, economic and social barriers contribute to hamper access and generate worse health outcomes(6). Among other weaknesses, health services operate in a fractional way in the healthcare system, which negatively affects patients and their families, who receive fragmented care(7).

The increasing complexity of treatment regimens associated with the growing prevalence of chronic conditions shows the importance of coordinating healthcare services. However, there is evidence that patients with multiple chronic conditions frequently do not receive patient- centered care, despite the importance of providing care that meets patients’ needs and preferences and the recognition of the role of patients and family members as part of the care team(8),(9),(10). This highlights the needs of planning strategies, mainly at the management level, that provide access in a timely manner and that guarantee comprehensive and continuous care, which in turn directly reflects on timely access to the proposed treatment for CRC patients.

Comprehensive care for people with CRC requires a demand for long-term care in order to ensure effective and integral care aimed at solving their health problems. Due to these characteristics, it is necessary that the care for cancer patients be carried out at home by family members and / or caregivers in addition to the specialized service.

A key component of integration in healthcare is care transition (CT), which is defined as “a set of actions designed to ensure safe and effective coordination and continuity of care as clients experience a change in health status, care needs, health care providers, or location”(11),(12). However, care transitions continue to be poorly managed, particularly from hospital to home(13). For patients with multiple, complex chronic conditions, such as CRC, CT is critical and strategies can be adopted to improve the quality in the care continuum(14).

Despite the evidence of unsafe care transitions, the impact of patient safety, and a plethora of literature on care transitions, significant gaps continue in practice and policy highlighting the need for implementation research to improve care transitions from hospital to community to advance quality and safety in our health care systems. Also, it is important to highlight that care transition strategies must include patients, families, and caregivers, once these folks are involved in the treatment and care. Furthermore, these patients are frequently dependent on technology and devices for continuity of care and support resources at the time of discharge(15).

From this perspective, to follow up on the patient’s care is necessary that the patient and their families are actively involved in discharge planning, as well as, their opinions be given visibility during this process(16). Thus, hospital discharge is a process that must be taken seriously in the patient’s care plan, with a view of facilitating their transition from the service to the home(17). In addition, to become effective, it is necessary that the time of discharge become a humanized process, providing support through professional guidance on care and self-care(18). Therefore, CT is characterized as a complex process that requires coordination and communication between people of different backgrounds, experiences, and skills(19).

CTis currently under construction and adaptationin Brazil. There is no standardization of strategies and effective actions to improve CT routinely performed during the hospitalization process. Thus, it lacks investigations at the national and international ambits(20),(16). In summary, conducting a bibliographic survey is essential to recognize the relevance and complexity of the hospital-community transition, especially in CRC patients. Thus, considering the relevance of identifying and summarizing safe, effective and resolving CT, this systematic review is justified.

The purpose of this systematic review and meta‐analysis is to evaluate the effectiveness of transition strategies from hospital-to-community care compared to usual care for patients with colorectal cancer to reduce hospital stay and reduce readmission and/or emergency room visits within 30 days. We seek to answer the following research question: How effective are care transition strategies from hospital-to-community compared to usual care for patients with colorectal cancer to reduce hospital stay, 30-day readmissions, and emergency room visits up to 30 days?

## Methods

### Protocol and registration

This systematic review and meta-analysis will be conducted according to the recommendations of the Preferred Reporting Items for Systematic Reviews and Meta-Analyses Protocols (PRISMA-P) 21. The protocol was registered in the International Prospective Register of Systematic Reviews (PROSPERO) under number CRD42020162249.

### Criteria for considering studies for the review

#### Types of studies

Will be eligible for inclusion, quasi-experimental studies or non-randomized trials and randomized controlled trials. Additionally, using the Population, Intervention, Comparison and Outcome (PICO) strategy we elaborated the guiding question of this review in order to ensure the systematic search of available literature: Which care transition strategies are effective compared to the usual care for the transition of CRC patients from hospital to community? The PICO strategy is provided in Table 1.

**Table 1:** Description of the PICO (population, intervention, comparator and outcome) strategy.

Definition	Description
P - Population	Colorectal cancer patients
I - Intervention	Care transition strategies
C - Comparison	Usual care strategies by the hospital healthcare team
O - Outcome	Effective care strategies/actions from the hospital to the community

This protocol will follow standard systematic review methods and use a random‐effects meta‐ analytic approach to synthesize the review findings. This review will consider ‘interventions’ as any specific initiatives intended to improve care transitions strategies from hospital to community for CRC patients in healthcare organisations.

#### Types of participants

In order to answer the research question studies must focus on strategies from the health care team developed from CRC patients.

### Data sources

#### Search strategy and data management

The following databases will be explored for relevant studies: PubMed/Medline, Embase, Cochrane CENTRAL and Lilacs. Languages to include: English, Portuguese and Spanish. Publication period: Studies published from inception until April 31st, 2020. In addition, we will review the reference lists of selected studies to see if there are other possible studies to be included.

The search strategy will be designed by combining keywords, MeSH terms, entry terms and using the AND/OR boolean operators. The following keywords and MeSH terms will be combined according to each investigated database: continuity of patient care, patient transfer, transitional care, patient discharge, patient readmission, care transition, transition of care, discharge planning, continuity of care, care coordination and colorectal neoplasms. This search strategy is described in Table 2.

**Table 2:** Search strategy for electronic databases.

PubMed/Medline	Embase
1. "Continuity of Patient Care"[All Fields]	1. Continuity of patient care/exp OR continuity of patient care
2. "Patient Transfer"[All Fields]	2. Patient transfer/exp OR patient transfer
3. "Transitional care"[All Fields]	3. Transitional care/exp OR transitional care
4. "Patient discharge"[All Fields]	4. Patient discharge/exp OR patient discharge
5. "Patient readmission"[All Fields]	5. Patient readmission/exp OR patient readmission
6. "Care transition"[All Fields]	6. Care transition/exp OR care transition
7. "Transition of care"[All Fields]	7. Transition of care/exp OR transition of care
8. "Continuity of care"[All Fields]	8. Discharge planning/exp OR discharge planning
9. "Care coordination"[All Fields]	9. Continuity of care/exp OR continuity of care
10. “Discharge planning”[All Fields]	10. Care coordination
11. 1 OR 2 OR 3 OR 4 OR 5 OR 6 OR 7 OR 8 OR 9 OR 10	11. 1 OR 2 OR 3 OR 4 OR 5 OR 6 OR 7 OR 8 OR 9 OR 10
12. "Colorectal neoplasms"[All Fields]	12. Colorectal neoplasms'/exp OR colorectal neoplasms
13. 11 AND 12	13. 11 AND 12
Cochrane CENTRAL	Lilacs
1. Continuity of patient care	1. "Continuity of Patient Care" OR "Patient Transfer" OR "Transitional care" OR "Patient discharge" OR "Patient readmission" OR "Care transition" OR "Transition of care " OR "Continuity of care" OR "Care coordination" OR "Discharge planning"
2. Patient transfer	2. "Colorectal neoplasms"
3. Transitional care	
4. Patient discharge	
5. Patient readmission	
6. Care transition	
7. Transition of care	
8. Discharge planning	
9. Continuity of care	
10. Care coordination	
11. 1 OR 2 OR 3 OR 4 OR 5 OR 6 OR 7 OR 8 OR 9 OR 10	
12. Colorectal neoplasms	
13. 11 AND 12	
	

Search results will be downloaded into Mendeley® data management software to identify and delete any duplicates. Full-text articles will be available for all authors.

### Eligibility criteria and quality assessment

Two authors will independently review the studies, in case of uncertainty/doubt, a third author will be involved in order to reach a consensus. The selection of studies will be performed in three steps. First, the titles will be read, followed by the abstracts reading and finally, the in- depth reading will be done, of the remaining studies from previous phases, in order to identify the potentially eligible ones.

To assess the levels and quality of evidence, studies will be graded as recommended by the Joanna Briggs Institute (JBI). Methodological quality will be assessed by different instruments according to the type of study. We will assess the risk of bias using the Cochrane Collaboration tool for randomized controlled trials and ROBINS-I tool for non-randomized trials studies of interventions.

### Data extraction and analysis

The narrative synthesis for each result will be provided in a table of evidence by two independent authors, which are going to reach a consensus in case of divergences. The following information will be extracted: article identification (authors/ year of publication/ country of study), study design, methodological aspects, focus of interventions and outcomes. Any relevant information missing from the study will be requested from the original authors of the article, if necessary. When appropriate, data will be combined and synthesized in a meta-analysis using Review Manager (RevMan 5.4) software. We will combine risk ratios (RR) or odds ratios (OR) for dichotomous data and mean differences (MD) for continuous data using random-effects models. All data will be presented with 95% intervals of confidence. Statistical heterogeneity between studies will be assessed using the I^²^ statistic.

This review will consider primary outcome as effective care transition strategies/actions from hospital to community. The secondary outcome of interest will be patient safety at hospital discharge; reduction of hospital costs to the health system. For the secondary outcome we will consider results of interventions that evaluated readmissions in colorectal patient, length of stay, and emergency department (ED) visits.

### Risk of bias in individual studies

Two reviewers will independently evaluate the quality of the studies. Possible disagreements will be resolved by consensus or with the consultation of third parties.

We will assess the risk of bias using the Cochrane Collaboration tool (RoB 2) for randomized controlled trials, using the following criteria or domains: bias arising from the randomization process; bias due to deviations from intended interventions; bias due to missing outcome data; bias in measurement of the outcome; bias in selection of the reported result. We will judge the risk of bias as ‘low risk’, ‘some concerns’ and ‘high risk’ as described in the Cochrane Handbook for Systematic Reviews of Interventions.

### Ethical considerations

This research is being conducted using meta‐analysis methods with existing trial data. The analyses will not include any identifiable patient data. Ethical committee approval was not required for this research.

## Discussion

Due to the lack of research on this topic in Brazil, this review will contribute to the practice and development of effective and safe care transition strategies from hospital to community for CRC patients. Therefore, development and implementation strategies during the discharge plan are essential for the safe transition of the patient from the hospital to the community(22). Thus, it is important that professionals in the hospital environment develop and offer CT for CRC patients and their families, with these transition strategies and practices based on evidence, with a view to contributing to greater patient safety, improving the continuity of care and, consequently, the quality of patients’ life(23) and the quality of health care(24),(25).

There is an expectation that this review will provide much needed evidence that effective care transitions could reduce short term hospital readmission, and may thus provide added value in the care of CRC patients.

The results of the review will be used to provide clear recommendations for hospital and primary care management to improve care transitions, and as a result, also improve integration in the healthcare system. Indeed, there is no previous research that aimed to find the most effective interventions to improve care transition in CRC patients. The key challenge is to find different interventions addressing all of the continuity of care dimensions. The outcome of this systematic review and meta‐analysis will be an understanding of the evidence for the relationship between effective care transition strategies from hospital to community for CRC patients.

### Limitations

Systematic reviews and meta‐analyses are powerful tools that summarize the evidence for current best practice guidelines for the available interventions for a particular problem(26),(27). However, scientific evidence from mega trials are rarely available for most medical conditions. Therefore, some limitations can compromise the quality of the review due to the inherently risk of bias associated with the eligible studies. To minimize it we will address each risk of bias with appropriate tools as described in the method section and will critically explore the evidence to provide recommendations using JBI method.

## Conclusion

CRC patients require ongoing care with the need for integration and organization of care across the health system, which must include their families and/or caregivers. Thus, arrangement must be done to provide actions in health services to ensure quality and safety during the care transition. The scientific evidence from this review will contribute to identify and disseminates effective strategies for the continuity of care for patients with colorectal cancer after hospital discharge. From this perspective, future studies research could also be developed in this field and contribute for the formulation and implementation of guidelines addressed to the continuity of care for patients with CRC.
